# Intra-tracheal multiplexed sensing of contact pressure and perfusion

**DOI:** 10.1364/BOE.442165

**Published:** 2021-12-03

**Authors:** Ricardo Correia, Brett Gadsby, Sergiy Korposh, Andrew M. Norris, Barrie R. Hayes-Gill, Rishie Sinha, Jonathan G. Hardman, David S. Gardner, Simon Talbot, Daniel Harvey, Julian McGlashan, Stephen P. Morgan

**Affiliations:** 1 Optics and Photonics Research Group, Faculty of Engineering, University of Nottingham, UK; 2 Division of Anaesthesia and Critical Care, Nottingham University Hospitals NHS Trust, Nottingham, UK; 3 Department of Anesthesiology, King Faisal Specialist Hospital and Research Centre, Riyadh, Saudi Arabia; 4 Department of Anaesthesia, Division of Clinical Neurosciences, University of Nottingham, UK; 5 Academic Unit of Injury, Inflammation and Recovery Sciences, School of Medicine, University of Nottingham, UK; 6 P3 Medical Ltd, Bristol, UK; 7 Department of Otorhinolaryngology, Nottingham University Hospitals NHS Trust, Nottingham, UK; 8 Centre for Large Animal Biotechnology, Sutton Bonington, University of Nottingham, UK; 9 These authors contributed equally to this work

## Abstract

Incorrect endotracheal tube (ETT) cuff inflation pressure causes significant problems for intubated patients. The technical development and first *in vivo* use of a smart ETT for measurements at the cuff-trachea interface during mechanical ventilation are described. The intra-tracheal multiplexed sensing (iTraXS) ETT contains integrated optical fibre sensors to measure contact pressure and blood perfusion. The device is tested during mechanical ventilation in a porcine model (N=6). For contact pressure, signals were obtained in all 30 measurements. For perfusion, data could be obtained in all 33 measurements. In the 3 cases where the cuff was inflated to an artificially high-level, blood occlusion is observed.

## 1. Introduction

Intubation is a necessary and common medical procedure. A patient’s airway must be secured to deliver oxygen to their lungs when their own ability to do so is compromised. This is particularly relevant when a patient is anaesthetized [1]. The cuff of a tracheal tube provides a seal in a patient’s airway, inflating to prevent air leaks around the cuff, as well as preventing aspiration of pharyngeal fluids [2]. Achieving an appropriate contact pressure between cuff and trachea is crucial: too high a pressure results in tracheal injury whereas too low a pressure results in micro-aspirations and lack of oxygenation of the patient.

In recent years, postintubation laryngotracheal injury cases have been documented [3]. Tracheal stenosis is a complication with long term effects on the quality of life of affected patients, as well as significant healthcare costs. Esteller-Moré *et al.* [4], reported an incidence rate of 4.7% for mild and severe subglottic and tracheal stenosis. However, there is a lack of good quality reports containing prospective follow up of intubated patients for 6 months with appropriate testing, meaning the actual incidence rate could be higher [5]. Tracheal stenosis is commonly caused by pressure necrosis and subsequent scarring due to excessive contact pressure of the ETT cuff [6]. Therefore, cuff pressure and mucosal perfusion must be monitored closely, with no current technology having the capability to do both. The recommended intracuff pressure (the pressure inside the cuff) is in the range of 20 to 30 cmH2O (1.96 to 2.94 kPa), ideally measured with a manometer, but often checked simply by squeezing the pilot balloon at the clinician’s discretion. Additionally, due to the fit of the cuff and asymmetry of the trachea, if accurate intracuff pressure measurements are performed, they can still differ from the true contact pressure exerted on the tracheal wall, allowing for over or under inflation. Su *et al.* [7], through use of Codman Pressure MicroSensors in a canine model, found that the ratio between cuff-trachea contact pressure and Intracuff pressure varied between 0.44 and 0.9 (with the intracuff pressure always being larger), for cuff sizes of internal diameter between 6.5 and 8.0 mm, with the 8.0 cuff size giving the closest match between pressures, and a smaller cuff size decreasing the ratio. Concerns about variation between these two pressures have been highlighted previously [8]. Furthermore, variations from patient to patient such as difficult airways caused by trauma, narrowing of the trachea, or obesity also impact the ideal cuff pressure for minimising risk of harm [9]. A lack of continuous or consistent measurements in a clinical setting could contribute to inaccurate and detrimental monitoring of the patient.

Under inflation of the cuff can also be harmful by causing a poor seal and loss of effective ventilation. Research has reported the association with increased rates of ventilator associated pneumonia (VAP), with simple continuous monitoring of intracuff pressure shown to reduce VAP cases by 12.1% compared to standard routine monitoring [10]. Critical care patients will develop VAP at percentages of 10-28%, with 86% of all hospital associated pneumonia being linked to mechanical ventilation [11,12]. The estimated increase in cost to treat VAP in the UK is £6,000 - £22,000 per patient [13]. Several US studies [14], have also estimated the increase in cost per patient to range from $ 10,000 – $ 40,000. Therefore, there is a clinical need to determine optimum tracheal-cuff contact pressure, while ensuring no tracheal ischemia.

An endotracheal tube (ETT) that has the capability to monitor both cuff contact pressure and tracheal perfusion is described here. The developed system, known as iTraXS (intra-tracheal multiplexed sensing), incorporates optical fibre sensors (OFS) for interface pressure and perfusion measurements into the cuff. Preliminary results have been described in two short conference papers [15,16]. A histological report on the tracheal injury following tracheal intubations and a survey on the professional attitude to smart tracheal tubes have also been described [17,18]. This paper describes the technical development and the first analysis of *in vivo* measurements.

Optical fibres are attractive as they are thin, flexible and resistant to electromagnetic interference. These sensors can be integrated into a standard ETT for measurements at the cuff-trachea interface, a cuff enclosed within a secondary double cuff configuration avoids direct contact between the OFS and tissue. There have been other attempts to integrate sensors into a tracheal tube [19,20], although none that combine contact pressure and perfusion. Photoplethysmography (PPG) of the body core, such as one obtained in the trachea, may be more desirable than a PPG obtained on the peripheries of a patient due to it being less affected by ambient light, skin tone and common medical complications such as blood loss, hypothermia and obesity [21]. Furthermore, a tracheal PPG could be used with cuff contact pressure to inform clinicians when tracheal ischemia is occurring. For vital sign monitoring, May *et al.* [19], implemented a pulse oximeter onto a flexible printed circuit board attached to the lumen of a tracheal tube. This system was able to acquire PPG signals in the trachea of all 10 anaesthetized patients. For contact pressure measurement, the Veneer PneuX is a medical device that measures cuff-trachea contact pressure, although cannot be integrated into a standard ETT, and does not measure perfusion [22]. Goethals *et al.* [20], attached an elastoresistant tactile sensor to the airway occlusion cuff to aid better tube placement, by leveraging the rings of cartilage in the trachea. To date, this approach has not been applied *in vivo*, or for perfusion measurements.

In this *in vivo* animal study with the iTraXS device, this paper primarily aims to establish whether contact pressure and blood perfusion data can be obtained through iTraXS in a porcine trachea. The relationship between intracuff pressure and the contact pressure exerted on the tracheal wall, as well as blood perfusion variation of the tracheal mucosa during varying cuff inflations is also reported.

## 2. Sensors

### 2.1 Contact pressure

**Fig. 1.**
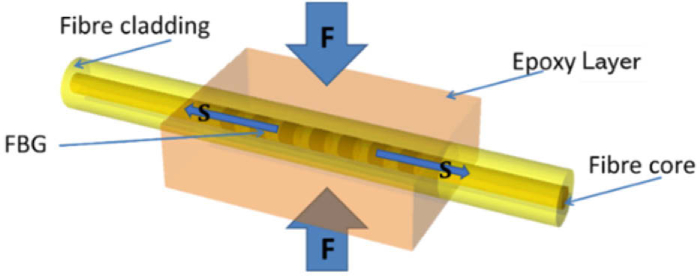
Fibre Bragg grating (FBG) is encased in a pressure transducing epoxy layer. A transverse force (F) applied orthogonally to the epoxy layer is transduced into a longitudinal axial strain (S) on the fibre, therefore changing the Bragg wavelength.

A fibre Bragg grating (FBG) was employed to measure contact pressure between the tracheal tube cuff and the trachea wall. An FBG consists of a periodic modulation of the refractive index [23] of an optical fibre (Fig. 1). This refractive index modulation will, at each grating plane, partially reflect a small amount of light due to the Fresnel effect, adding up in the backward direction whenever constructive interference occurs due to the satisfaction of the Bragg condition. This results in the reflection of a narrow bandwidth of light. The Bragg wavelength ( $\lambda_{Bragg}$ ) of an FBG is defined in Eq. (1),

(1) $$\lambda_{Bragg}=2n\Delta$$

where n is the effective refractive index, and Δ is the grating period.

Due to the dependency of the effective refractive index and grating period on temperature (T) and strain (S), the Bragg wavelength will shift as a function of these two parameters [24]. The change in Bragg wavelength due to strain and the thermal effect is given by Eq. (2),

(2) $$\frac{\Delta \lambda_{Bragg}}{\lambda Bragg}=\left(1-p_{e}\right)S+\left\{\left(1-p_{e}\right)\alpha +\zeta \right\}\Delta T$$

where all constants relate to the fibre, $p_{e}$ is the photoelastic constant, α is the thermal expansion coefficient, and ζ is the thermal optic coefficient. The longitudinal strain effect is described by the first term of Eq. (2), and the thermal effect, which is a convolution of the thermal expansion and thermal optic effect of the material, is described by the second term of Eq. (2).

Although an FBG is a highly sensitive strain gauge, it needs to be adapted to make it sensitive to contact pressure. This can be achieved by encasing the FBG in an epoxy layer that transduces transverse pressure (F) into an axial strain (S) along the FBG due to the deformation of the layer [25], as shown in Fig. 1. A range of materials could have been used to surround the fibre and transduce a strain into pressure in different manners, although the epoxy was chosen for its simplicity, elastic properties, it being easily cured, and its biocompatibility. It is worth noting that materials of different Young’s moduli can be used to change the sensitivity of the pressure sensors.

### 2.2 Blood perfusion

Photoplethysmography (PPG) is used to non-invasively measure the change in blood volume of microvascular tissues and is performed either in transmission or reflectance mode [26]. Transmission mode is generally limited to the extremities of the body such as a finger or ear, whereas reflection mode is more versatile and is the preferred modality for a tracheal PPG. As discussed in the introduction, a core PPG may be more desirable to clinicians than a peripheral PPG.

A PPG signal consists of a background (DC) signal originating from tissue properties such as scatter and components due to respiratory movements, the nervous system, and metabolic processes. The other important constituent of a PPG signal is a pulsatile AC component attributed to blood volume changes, which is synchronised with the cardiac cycle. The pulsatile signal due to the cardiac cycle is often extracted by filtering of the raw, wide bandwidth blood volume optical intensity signal to reveal the AC component of the PPG. The PPG signal can be measured with two optical fibres, light from one fibre illuminates an area of tissue where it is scattered and absorbed by blood in the capillaries and arterioles. A second optical fibre, also in contact with the tissue, and typically 3-5mm away, collects the reflected light resulting in the raw PPG signal. The associated light source and photo-detection circuitry is discussed in Section 3.3. The ratio of the pulsatile AC component and the non-pulsatile DC component expressed as a percentage is referred to as the perfusion index (PI) and is an indication of the magnitude of blood flow, i.e. perfusion [27].

## 3. Methods

### 3.1 ETT fabrication

**Fig. 2.**
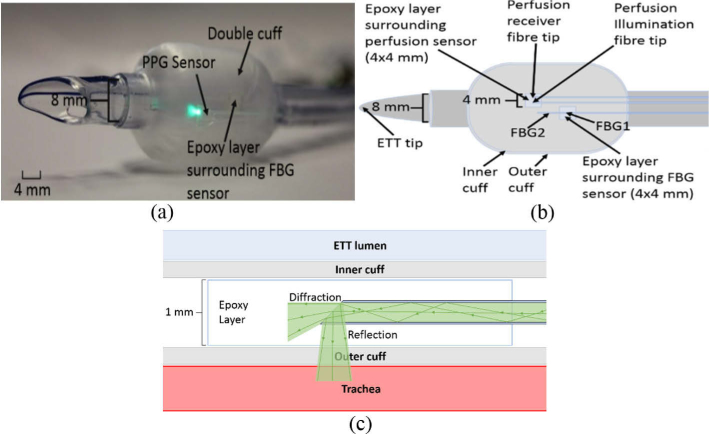
iTraXS tube illustrating the double cuff housing the PPG sensor and FBG epoxy layer that measures tracheal perfusion and contact pressure respectively. (a) Photograph. (b) Schematic (fibres in blue placed between inner and outer cuff). (c) Schematic of the illumination fibre in the perfusion sensor showing how it fits between the cuffs of a double cuff ETT, and how the light reflects into the trachea.

A Mallinckrodt cuffed ETT (107-80, Covidien, UK) with the standard high volume, low-pressure (HVLP) cuff made from polyvinylchloride (PVC) was used, this allows for a more low-pressure seal in the trachea. The ETT was modified with a dual cuff to provide a safe cavity for fibre optic measurement capability. The contact pressure and perfusion monitoring fibres are enclosed between the inner and outer layer of the cuff of a commercially available, standard size 8.0 and 9.0 mm ETT, where the size refers to the internal diameter of the tube in mm. The outer cuff has the same shape and material as the inner cuff (HVLP, PVC), with the only difference being its larger size. These sizes were chosen so that the ETT would fit the tracheas of the subjects, but it is worth noting that iTraXS can be incorporated into any standard ETT. By integration into the cuff, the sensors are held close to the trachea whilst avoiding direct tissue contact. All fibres were placed in the cuff such that they are on the inside (Magill) curve of the ETT (Fig. 2). In the event of any breakage, the sensors will remain within the cuff due to the double cuff arrangement and not be inhaled by the subject.

### 3.2 Contact pressure and perfusion sensor

FBGs of length 3 mm were written along the core of a highly photosensitive optical fibre (PS1250, Fibercore, UK). An in-house fabrication system, consisting of a frequency quadrupled Nd:YAG laser emitting at 266 nm (Minilite, Continuum, USA) using a Talbot interferometer via a 1072 nm period phase mask (Ibsen Photonics, Denmark) was used, producing two FBGs with Bragg wavelengths of 1551 nm and 1556 nm. Estimates of the grating periods of the FBGs were found by using the Bragg relation (Eq. (1)) with an effective refractive index of the fibres, supplied by the manufacturer as 1.4441. For the 1551 nm and the 1556 nm fibres, grating periods of 537 nm and 539 nm were found, respectively. However, due to the process of the fibres being made highly photosensitive by the manufacturer, the effective refractive index is an estimate due to batch-to-batch variation. The FBG’s reflectivity was approximately 70%, giving FBG losses of approximately 30%. The Bragg wavelengths of 1551 nm and 1556 nm were chosen due to opto-electronic components being widely available in the C-band (1530–1565 nm). Furthermore, the two wavelengths used are sufficiently separated that the optic interrogator can distinguish them in the presence of wavelengths shifts due to a range of contact pressures. The FBG positioned closer to the distal end (FBG2 – 1551 nm) was used to compensate for temperature changes and was left unfixed at its end, while the other FBG (FBG1 – 1556 nm) was embedded into a 4 × 4x1mm epoxy layer (Fig. 2). The epoxy in which FBG1 was embedded was Norland Optical Adhesive 65 (Norland Products Inc, USA), having a Young’s modulus of 138 MPa, and was cast using a mould made from polytetrafluoroethylene (PTFE). In addition to increasing the pressure sensitivity of the FBG, the epoxy layer also provided the FBG with better durability.

The calibration methodology for characterising the relationship between the temperature compensated Bragg wavelength shift and the pressure for the FBG epoxy layer has been described previously by Liu *et al.* [28], in which known loads are applied using an in-house manufactured actuator to each FBG sensor to obtain a force vs Bragg wavelength shift relationship. The applied force is then converted into an applied pressure using the area of the layer. However, it was found that the relationship between the applied force and transduced pressure is not simply found using the top surface area of the epoxy layer. Therefore, a calibration factor was generated for each FBG sensors using the *in vivo* experimental data, such that the average manometer intracuff pressures are equal to the average FBG contact pressures. This allows us to investigate how the contact pressure differs from the intracuff pressure at different positions in the trachea.

Two plastic optical fibres (Poly (methyl methacrylate) (PMMA), Asahi DB-500) with core refractive index 1.49 and diameter 0.5 mm were used to acquire the PPG signals. The illumination fibre’s distal end was cleaved at ${\text{45}}^{\circ }$ with a sharp blade, allowing for an increase of > 16% light to be reflected perpendicular to the surface of the cuff and into the tracheal tissue (see Fig. 2(c)). The fibre delivers an optical power of 5.1 mW at a wavelength of 530 nm, well below the threshold to cause tissue damage as per the photobiological standard BS 62471 where the maximum permissible exposure is 200 mW. The reflected light was received by a second optical fibre which was also cleaved at ${\text{45}}^{\circ }$ to assist with the collection of light and was separated from the illumination fibre by 2-3 mm. This separation was chosen as it was within the ideal range for obtaining PPG signals [29], whilst minimizing this distance allows for a smaller sensor region and maintains the elastic properties of the cuff.

### 3.3 Opto-electronic measurement unit

**Fig. 3.**
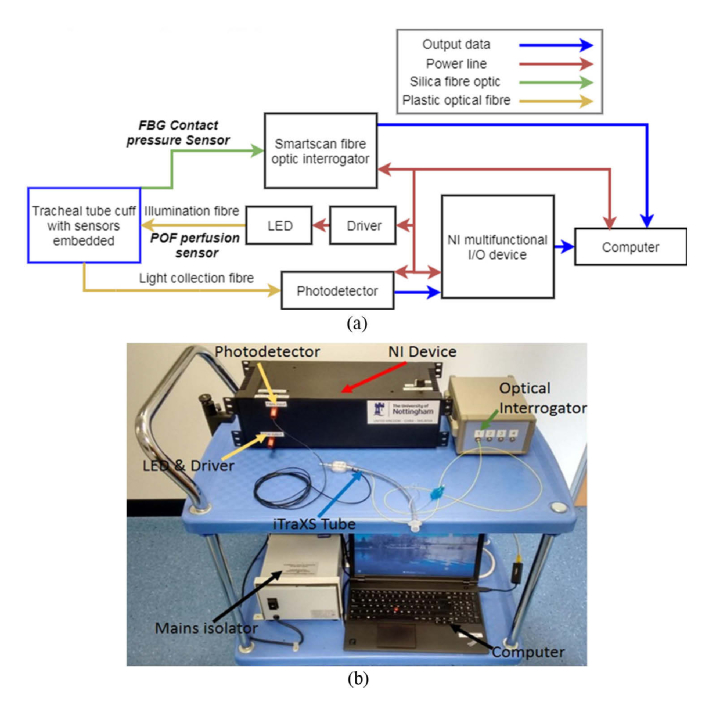
(a) Instrumentation design for measurement of the FBG pressure sensor and the optical fibre perfusion sensor responses (NI – National Instruments, LED – light emitting diode. POF – plastic optical fibre, FBG – fibre Bragg grating). (b) Photograph of the scientific and medical equipment used in the operating theatre.

Figure 3(a) shows a block diagram of the opto-electronic instrumentation developed. The FBG contact pressure sensor was connected to an OEM fibre optic interrogator (Smartscan, Smartfibres, UK). The scanning frequency of the interrogator was 2.5 kHz per channel, over the wavelength range 1528-1568 nm, with a resolution of 16 pm and a repeatability of <1 pm. The interrogator was connected to a laptop to store the pressure data via an ethernet cable.

The perfusion sensors’ illumination was generated by a fibre-coupled high-power LED (M530F1, Thorlabs, USA), with central wavelength 530 nm, yielding a full width half maximum of 33 nm and an optical power output of approximately 5.1 mW. The LED was driven with a maximum output of 1000 mA and a current ripple of 8 mA (LEDD18 driver, Thorlabs). The collection fibre was connected to a photodetector with an integral trans-impedance amplifier (PDA36A-EC, Thorlabs), having a switchable gain set to 60 dB. All connectors are SubMiniature Version A (SMA). The analogue-to-digital converter (ADC) channel of a data acquisition card (National Instruments, USB-6361, USA) was used to acquire the output of the photodetector with a sampling frequency of 5 kHz and 16-bit resolution, operating with a 5 V power supply, and 5 Hz anti-aliasing filter.

### 3.4 Experimental protocol

The pre-clinical investigation was established to assess primarily whether optical sensors could be used to measure cuff-trachea contact pressure and to obtain tracheal PPG signals. Further secondary objectives were to study the relationship between intracuff and contact pressure and to investigate how blood perfusion varies with cuff inflation pressure (intracuff pressure).

Ethical approval was provided by the University of Nottingham and under Home Office license (PPL 40-3410). The subjects were ventilated with a veterinary anaesthesia ventilator (Matrx 3000, Midmark) set to a typical volume cycled ventilation mode. Heart rate was monitored by an electrocardiograph (ECG), although this was used only by clinicians and the data was not stored. iTraXS tubes (size 8.0 mm) were used to secure the airway and allow mechanical ventilation of the lungs in a total of 6 pigs (Canberra 12 mixed breed; Landrace/Large White/Duroc), with ages 10 to 12 weeks, and mean weight 60.6 ± 5.0 kg, undergoing surgery for alternative non-recovery experiments. The pigs are referred to as subjects 1-6 throughout the remainder of this paper. The subjects were fasted for 24h and sedated with ketamine (5 mg/kg), intramuscular buprenorphine (0.05 mg/kg) and detomidine (0.1 mg/kg). After 15 to 20 minutes of sedation, the larynx was further relaxed with alfaxolone (0.7-2.4 mg/kg) given intravenously, and the iTraXS tracheal tube was inserted into the trachea following standard intubation procedures. The subjects were then stabilised and placed under general anaesthesia, maintained with isoflurane (1-2%) mixed with oxygen and air, maintaining over 94% oxygen saturation. Tidal ventilation was started in the range of 10 to 12 ml/kg and adjusted to maintain an end tidal carbon dioxide (ETCO2) of 6 to 8 kPa.

**Fig. 4.**
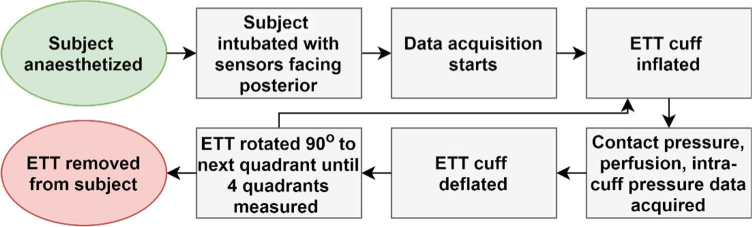
Experimental flow chart showing an overview of the experimental procedure.

The tube was initially placed so that the sensors were positioned towards the posterior tracheal wall (side closest to the oesophagus) and the cuff then inflated. Signals from each quadrant of the trachea were then obtained by deflating the cuff, followed by a rotation of the whole tracheal tube by 90°, and then inflating the cuff. In this way the sensors were positioned towards the posterior, left lateral, right lateral and anterior of the trachea respectively. For each rotational orientation, the cuff was inflated to an intra-cuff pressure between 20-80 cmH2O (1.96-7.85 kPa), as measured by an inline manometer, and then maintained for several minutes. In certain positions the cuff was also over inflated to pressures exceeding 300 cmH2O, so to investigate the measurement of severely diminished blood perfusion in the microcirculatory mucosa bed. It should be noted that pressures are represented in units of cmH2O throughout this paper as this is applied in clinical practice. Recordings were stored on a Windows based laptop and used for subsequent analysis. An overview of the experimental procedure used can be seen in Fig. 4.

Analysis of the data was performed using MATLAB ver. R2019b. The sampling rate for the manometer intracuff pressure was 1 s, the fibre contact pressure was 0.4 ms, and the fibre perfusion probe was 0.2 ms. A moving average filter of 75 points and 25 points was applied to the perfusion and contact pressure measurements respectively in order to remove some of the white noise above 70 Hz, since PPG signals generally range between 0.5-5.0 Hz [30], this filter was chosen to reduce noise but not distort the PPG. The moving average filter is calculated by use of a sliding window across neighbouring elements, if there was an odd number of data points the window is centred about the middle of the data, the window is also truncated at the ends where there are insufficient data points to fill it, so the average is only taken over the data that fills the window. The filter has a zero-phase degrees response by processing the data in the forwards and backwards direction.

## 4. Results

Examples of pressure and perfusion signals are presented with further investigation of the relationship between intracuff and contact pressure; and perfusion with either intracuff or contact pressure. Finally, a tabulated summary draws together the data acquired. Each data acquisition ranged from 200-3000 s, however, the acquired signals were consistent throughout this range and so 200 s sections were used in all cases so that longer data sets do not more heavily influence the analysis. The total data used for each subject can be seen from Table 1 (Section 4.3). The 5th and 6th pressure FBGs were damaged during experimentation and produced no usable contact pressure measurements for reasons described in the discussion section.

**Fig. 5.**
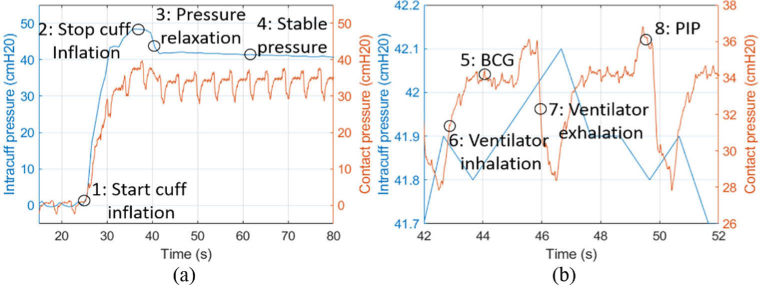
Intracuff pressure measured by an inline manometer (blue) versus cuff-trachea contact pressure measured by an FBG (red) for subject 1, sensors at the posterior of the trachea. (a) The initial inflation and resulting pressures. (b) a 10s section of (a) after the cuff was fully inflated. 5: BCG refers to a ballistocardiograph response, explored further in the discussion (Section 5.1).

### 4.1 Cuff-trachea contact pressure

An example of the measured intracuff and contact pressure during a cuff inflation is shown in Fig. 5 (subject 1, sensors at posterior). The increase in intracuff pressure was taken as an absolute increase from an initial fully deflated cuff. The FBG contact pressure was found by converting the temperature compensated Bragg wavelength shift into a pressure with a corrected sensitivity as described in Section 3.2. The ventilator response strongly influences the contact pressure as can clearly be seen from Fig. 5(b). Several features can be observed in Fig. 5. At Point 3, the pressure relaxation refers to a small decrease in pressure, likely caused by the settling of the cuff in the trachea post inflation and the slight change in elasticity of the cuff due to temperature. At Point 5, the BCG highlights an apparent single ballistocardiograph response, and can be seen throughout Fig. 5. However, this feature is not analysed in this paper and will be an area for future research. At Point 8, PIP refers to the peak inspiratory pressure, with the peak at the end of the ventilator likely being an artefact of the ventilator producing a positive pressure just before exhalation, or back pressure from the lungs.

The intracuff pressure in this example was found to be ( 42±2 ) cmH2O, whereas the contact pressure measured was found to be ( 33±3 ) cmH2O. A slight drift in manometer pressure that equates to approximately 0.5 cmH2O a minute can be seen, this is occasionally present throughout the data and most likely originates from a small loss of pressure in the seal of the cuff/manometer interface, the trachea stretching slightly as a constant pressure is exerted on it, or small cuff elasticity changes due to temperature.

The pressure variation due to ventilation that was registered by the FBG contact pressure sensor follows the pressure changes inside the cuff measured by the manometer. A typical volume cycled ventilation mode can be seen in Fig. 5(b), where the ventilator induces a contact pressure change of ∼ 9 cmH2O, caused by a prespecified volume of air being delivered to the lungs, irrespective of resistance. The ventilator pressure response is consistent across orientations and subjects.

**Fig. 6.**
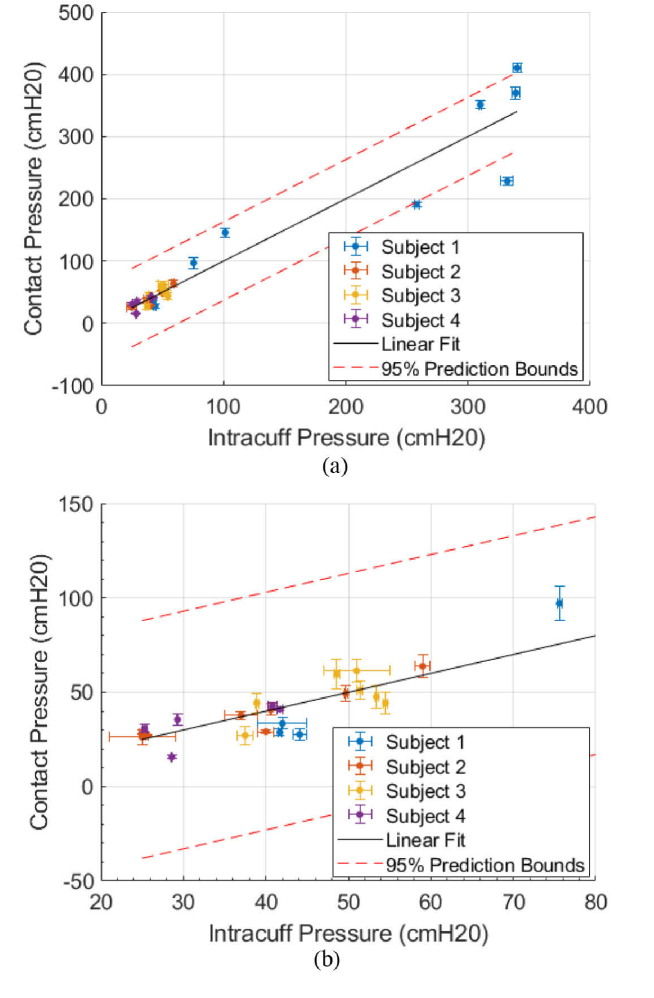
Pressure measurements for all quadrants. (a) The relationship between the intracuff pressure of a tracheal tube cuff and the FBG contact pressure of the cuff trachea boundary for Subjects 1:4. (b) a close up of the data in (a), showing only the more densely populated, medically relevant, low-pressure region (20-80) cmH2O.

In Fig. 6, each “cuff inflation”, beginning at the stable section after the pressure relaxation (after Point 3 in Fig. 5(a)) is plotted against the measured tracheal contact pressure. A contact pressure measurement could be obtained from 30 out of 30 of the cuff inflations where the FBGs were undamaged. A 200 s window after the cuff inflation was averaged to provide the final pressures. Baselines for the temperature and pressure FBGs were produced for each set of data taken by intubating the subjects with the iTraXS ETT and then averaging the continuous data pre-inflation. Due to the use of the experimental data itself to calculate the contact pressure sensitivity, a linear model predicts a relationship between intracuff pressure and contact pressure across all measurements of 1 ± 0.1, with high correlation (R-squared = 0.9227) for 30 data points.

**Fig. 7.**
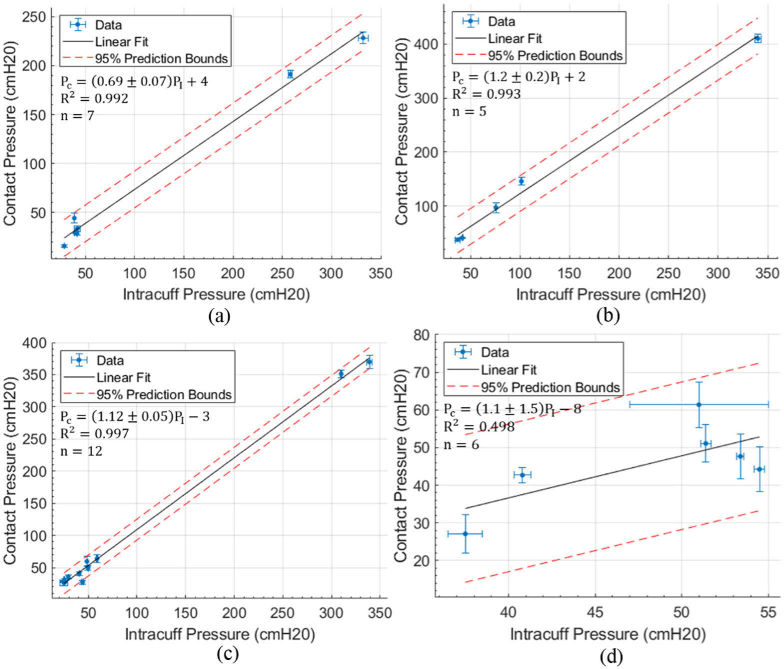
Intracuff pressure and cuff-trachea FBG contact pressure relationship for subjects 1:4, separated by trachea quadrant for the (a) posterior, (b) left lateral, (c) right lateral and (d) anterior. $P_{C}$ is the contact pressure of that quadrant, $P_{I}$ is the intracuff pressure of that quadrant. The prediction bounds (95% confidence interval) are given on the gradients of the linear models, along with the R-squared values ( $R^{2}$ ) and number of data points (n).

Figure 7 contains all the contact pressure measurements across all subjects where the fibres were not damaged (subject 1:4), separated into each quadrant. The contact pressure and intracuff pressure ratio between the left lateral and right lateral side of the trachea agree within the 95% prediction intervals (1.12 ± 0.05 compared with 1.2 ± 0.2). However, the pressure ratio for the posterior of the trachea is lower (0.69 ± 0.07). There is on average 40 ± 10% less contact pressure exerted on the posterior of the trachea than the left lateral and right lateral sides for the same intracuff pressure on average across all subjects, likely due to the softer tissue at the posterior. The anterior of the trachea produces a weakly positively correlated relationship of 1.1 ± 1.5.

### 4.2 Tracheal blood perfusion

In total, 33 out of 33 of the possible PPG data sets were extracted using peak detection from the tracheal orientations, producing a total of 637 PIs across all subject and orientations, when the PPGs were appropriately windowed as described later in this Section. For 3 PPGs, a PI = 0 was produced when the manometer pressure exceeded 337 cmH2O and the tissue was ischaemic and is also described later in this section. Data acquisition ranges from 200-3000s, therefore a 200s segment of each inflation is used to generate the PPG data for each recording. If a PPG signal was obtained then it remained for the duration of the data acquisition, apart from small movement artefacts accounting for < 5% of all usable data.

**Fig. 8.**
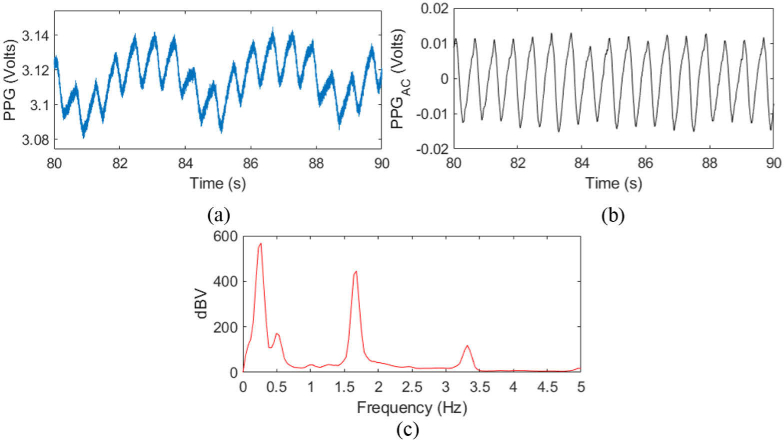
A 30s duration PPG signal from tracheal mucosa. (a) Unfiltered PPG signal. (b) PPG signal with low frequency content removed. (c) Fourier spectrum of the unfiltered PPG signal, where the heart rate is ∼1.6 Hz (96 bpm), with the 0.15-0.47 Hz components from the ventilator.

An example PPG signal for subject 1 can be seen in Fig. 8(a), where the sensor was placed towards the posterior of the trachea. Three components can be observed – the PPG, lower forced ventilator frequency and higher frequency noise in the trace (Fig. 8(a)). Detection of the ventilator signal was easily observed by Fourier analysis of the filtered data as shown in Fig. 8(c). Here, the frequency range of the ventilator was found to be 0.15-0.47 Hz, which corresponds to the physical ventilation rate of approximately 4 s – the variability is caused by variations in the forced ventilator frequency. Removal of this ventilator signal allows for a more accurate perfusion AC signal to be extracted from the PPG signal (Fig. 8(b)). The pulse rate can be seen in the FFT to be ∼96 bpm. Filtering of the data was performed by a 0.66 Hz first-order, finite impulse response (FIR) high-pass filter, with stopband attenuation of 60 dB and time delay compensation. The passband corresponds to the upper limit on contributions from cardiac signals (1.5 s), therefore removing some of the low frequency ventilator characteristic. Perfusion data was used to generate a perfusion index for 33 intracuff pressures (all usable data) from the time domain signal, ranging from 25-350 cmH2O.

**Fig. 9.**
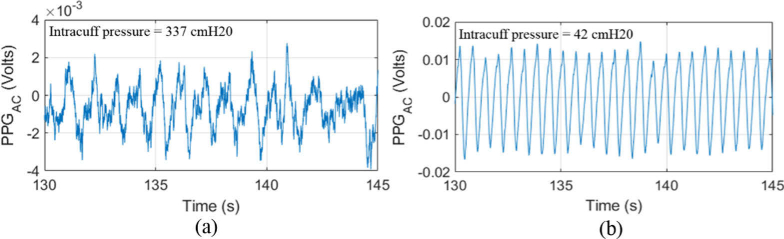
(a) Tracheal PPG signal after 75-point averaging as well as filtering to remove ventilator signal and DC voltage, at an intracuff pressure of (a) 42 cmH2O and (b) 337 cmH2O. The PPG amplitude and PI decreases with an increasing intracuff pressure.

Figure 9 shows an example of the AC amplitudes of the PPG data at inflation pressures of 42 cmH2O (Fig. 9(a)) and significantly exceeding guideline pressure of 337 cmH2O (Fig. 9(b)). The measurements were taken sequentially from subject 1 at the same position and orientation (posterior). At the lower inflation pressure (42 cmH2O, Fig. 9(a)) the AC (pulsatile component) can be clearly observed with an amplitude of ∼0.024 V, which results in a PI of ∼1%. At very high inflation pressures (337 cmH2O, Fig. 9(b)) the pulsatile AC component is almost completely removed from the signal and the PPG is dominated by the residual ventilator signal, for this and all other cuff inflations exceeding 337 cmH2O the AC amplitude of the PPG signal produced a PI that was lower than 0.02% as well as containing significant noise such that peak detection was unable to produce values and results in a PI = 0.

**Fig. 10.**
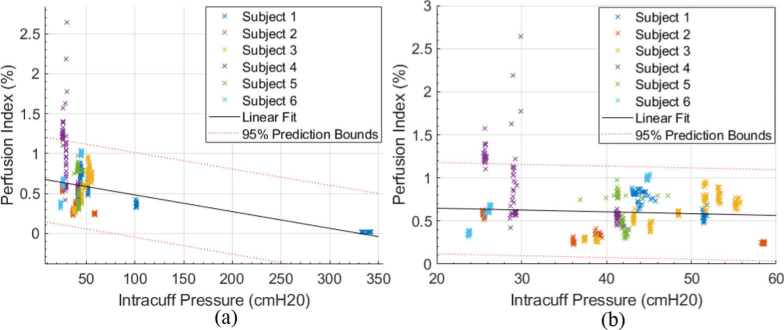
Tracheal perfusion index variation with tracheal tube intracuff pressure for Subjects 1:6. (a) whole range. (b) More clinically relevant range of 20:60 cmH2O.

Aggregated data of intracuff pressure and perfusion index across all subjects and sensor positions are shown in Fig. 10. Beat-to-beat AC values were found using peak detection with a minimum peak prominence threshold which corresponds to a PI > 0.02%. Peaks also had to be more than 0.15 s apart and there had to be at least 10 peaks every 12 s for a PI to be generated. A single PI was generated from a moving window of 12 s followed by a gap of 1 s, where the median of the beat-to-beat AC amplitudes was used, and the mean of the DC values was used. No strong relationship can be seen in this case, or between contact pressure and perfusion in the region most of interest. A linear model predicts a relationship of -0.0021 ± 0.0004% per cmH2O with an adjusted R-square of 0.152.

### 4.3 Summary of results

It is worthwhile investigating subsets of the data for different subjects and different positions. A summary of the relationships between the different parameters studied is shown in Table 1. A linear fit was generated for each data set. 8/8 of the contact pressure vs intracuff pressure data are positively correlated. 7/10 of the perfusion index vs intracuff pressure relationships correlate negatively. 6/8 of the perfusion index vs contact pressure data shows that an increase in contact pressure leads to a decrease in perfusion index, for the data separated by subject and tracheal quadrant. It is worth noting that the perfusion values are weakly correlated with pressure so care must be taken in over interpreting the data.

**Table 1.** Summary of contact pressure, intracuff pressure and perfusion index data

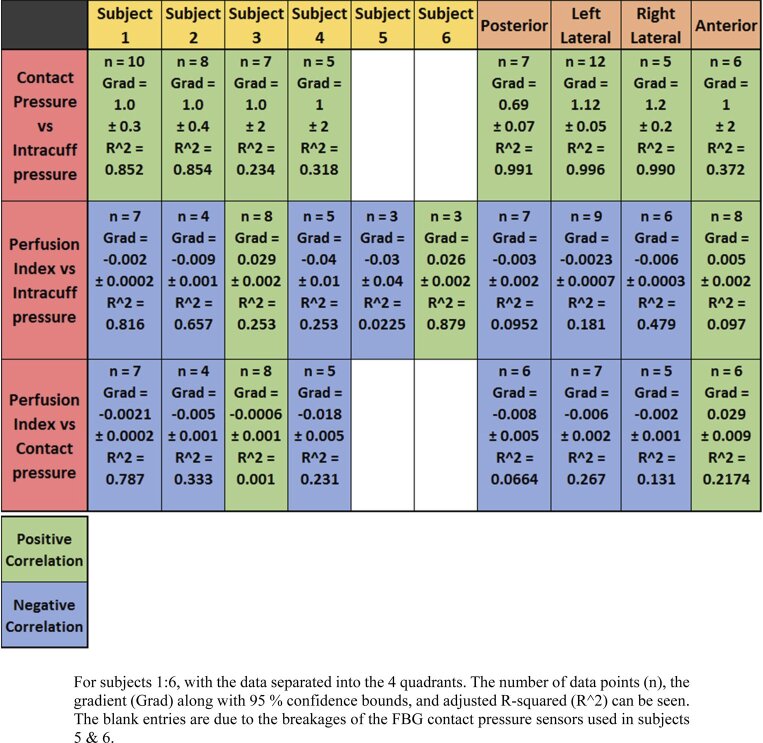

## 5. Discussion

The aim of acquiring preliminary measurements in an *in vivo* porcine model using the iTraXS system with a range of orientations and sensor positions, across multiple subjects, was achieved. The relationships between intracuff pressure, contact pressure and perfusion index have been investigated as secondary objectives. The FBG contact pressure sensor correlates with the intracuff pressure and is dependent on orientation in the trachea. The perfusion sensor shows its ability to reliably measure perfusion index. Two of the six contact pressure sensor fibres experienced breakages but the total number remaining was 30 pressure measurements and 33 PPG measurements showing high potential for follow up work. These breakages were due to not recoating the FBG after laser inscribing and relying on the epoxy layer alone to reinforce the structure. This can be resolved in future by either recoating the fibre [31] before embedding in the epoxy layer or by utilising FBGs inscribed by a femtosecond laser. The measurements have been made with a Mallinckrodt cuffed ETT with a high-volume, low-pressure cuff. However, with additional characterisation the method is compatible with any cuffed ETT.

### 5.1 Contact pressure

The ideal cuff pressure is one which sufficiently seals the patient’s airway whilst not causing damage to the tissue. Throughout the results there is some variability between intracuff pressure and contact pressure, which appears to be dependent on rotational orientation as can be seen from Fig. 7, although, due to a lack of imaging of the exact placement of the sensors in the trachea, this will need to be further investigated. However, this research leads to a better understanding of how the cuff contact pressures are exerted on the trachea and could lead to an improved appreciation by clinicians of the development of tracheal injury and VAP due to measurements of the local contact pressure.

The anterior of the trachea provides low correlation between intracuff pressure and contact pressure. This potentially means that the sensor performs poorly in this quadrant, or the contact pressure greatly differs from the intracuff pressure due to the asymmetry of the trachea, curvature of the tube, and it being rotated 180° to its normal position. Furthermore, from Fig. 7, the posterior of the trachea produces on average 40 ± 10% lower contact pressure than the left and right lateral sides, likely due to the softer tissue of the posterior. These variations highlight the need for a more sophisticated tracheal cuff, one where the local contact pressure can be accounted for, potentially resolved by multiple pressure sensors that can account for the variability of the contact pressure. Another potential source of signal variation is the presence of rings of cartilage in the trachea. Cartilage rings create D-shaped prominences of relatively stiff tissue at intervals of a few mm along the length of the trachea. A sensor may be positioned directly over a cartilage ring and cause a higher pressure reading. This could be resolved by increasing the number of sensors or implementing a larger sensor that straddles the cartilage rings. Further work is also required in sensor manufacturing due to each sensor being handmade and individually calibrated. It should be noted that limited data points and range, especially at large intracuff pressure, makes for a weaker statistical analysis. There are also two distinct groups of pressures in Fig. 7(a)-(c), separated by a pressure of greater than 200 cmH2O which could affect the measured relationship between intracuff and contact pressure, and the R-squared values. In future studies, a more even distribution of pressures should be used. Although the data is physically anticipated, it suggests that further study is required.

A ballistocardiograph refers to the ballistic forces produced by the heart and circulation of blood around the body. Typically measured non-invasively from the surface of the body and can be seen in Fig. 5. This is a new type of signal measured at the cuff trachea interface and further research is required to verify and validate this feature. However, there is potential to use this as an indication of pulse pressure and heart function. The interpretation of pulse pressure and volume variations is a large field, encompassed in non-invasive cardiac output measurements typically from PPG signals, but also derived invasively from direct arterial cannulation. It is also possible to validate that the BCG signal originates from the heart rate due to it having the same frequency as one derived from the PPG. There is an interest in how these circulation measures vary during mechanical ventilation, if the circulation is relatively poor, e.g. due to blood loss, then the heart’s performance is more affected by positive pressure in the chest. Invasive measures are more reliable but can be challenging due to associated risks. Furthermore, a PPG derived peripherally may not precisely reflect central perfusion, giving tracheal force signals a greater potential to improve clinical capabilities when used in conjunction with a core PPG.

The pressure changes due to ventilation that can be seen in the FBG contact pressure (Fig. 5) could provide useful information about the ventilator pressure and the response of the trachea and lungs to this pressure. The effect of ventilation on the cuff-trachea interface has been scarcely reported in literature and will be an area of further study. Typically, peak inspiratory pressure (PIP) occurs just before the ventilator cycles. However, in Fig. 5 the PIP sharply spikes at the end of the cycle. The pressure spike could be explained by back pressure travelling up the trachea from the end of the ETT and from the lungs. However, this spike could also be explained by the tracheal wall initially bulging during the low-pressure stage and then stiffening as it reaches its limit of elasticity. It should be noted that this study typically found that the FBG contact pressure traces when the sensors were placed towards the posterior to be less affected by the forced ventilator pressures, which supports the posterior being more elastic than the lateral and anterior, and will be explored in future work.

### 5.2 Perfusion

Core PPG performed in the trachea could be significantly more desirable to clinicians than a peripheral PPG, due to it being less affected by common medical complications such as blood loss and hypothermia [19], [21]. Apart from the cases where the cuff pressure was raised to a high value outside the clinical range, PPG signals could be obtained in all cases. The reliability can be attributed to the positioning of the PPG sensor at the cuff-trachea interface ensuring that it is always in intimate contact with the trachea, thus making it relatively immune to motion artefacts. The tracheal perfusion indexes are typically in the range of 0.2–1% in this work. There is, however, no widely accepted standard for peripheral PIs and they are dependent on physical location and wavelength used, but they typically range from 0.02–20%, with reported averages of ∼1.4% [32]. Although the values are on the lower end of this range, it is mostly attributed to shunting effects from a large DC component in the trachea, with the pulsatile AC component generally being strong. This can be addressed by shielding the two fibres from each other in future work to avoid direct coupling of light from source to detector fibre. Cartilage rings could also play a role where the rings could mask blood vessels. The presence of the ventilator signal in the PPG makes calculating reliable PIs challenging. By filtering the PPGs some of the ventilator signal can be removed, although it is still present in both the AC and DC components. In future work, the ventilator signal should be isolated so it can be more easily removed, such as by use of a more modern ventilator where the ventilator pressure signals can be directly output from the ventilator. The quality of the PPG varied between experiments although could always be obtained at intracuff pressures below 337 cmH2O, this can likely be explained by the composition of the trachea. A stronger PPG is obtained when the sensor is placed against a muscular section of the trachea, whilst a slightly poorer signal is achieved if the sensor is placed upon a ring of cartilage. However, due to the c-shape of the rings, a rotation to one of the three other orientations always presented with an improved PPG. This is the same effect as a PPG measured in contact and non-contact where the PPG amplitude is greatly diminished in non-contact [33]. Hence, there are several causes of a varying AC and DC PPG components occurring that can further disturb the PI.

In the future, a cuff with at least 3 sensors covering the posterior, left and right lateral would greatly improve reliability. Although no clear statistical relationship is observed between cuff pressure and perfusion index across all subjects, there are instances of blood occlusion to the microcirculatory tracheal mucosa observed which supports future *in vivo* investigation. Similarly, there are specific cases of blood perfusion returning as pressure is relieved. These findings could help aid clinicians in preventing tracheal ischemia. A broader study is required to monitor the accuracy of these findings, as well as to investigate the clinical benefit of these signals.

The unique cartilage ring structure of the trachea has been postulated to be potentially problematic for both pressure and PPG measurements, as well as measurements made at the anterior. Future research will need to account for this by implementing more optical fibre sensors to cover multiple positions, allowing for simultaneous measurements and avoiding tube rotation to investigate this problem. A more complex or larger FBG epoxy layer that could straddle the individual rings could also avoid this issue. Multiple FBG sensors coupled with an elongated illumination region by removing the cladding could also help to improve PPG signal reliability.

## 6. Conclusion

The technical development of the iTraXS smart endotracheal tube has been described along with its first implementation in a porcine model. Contact pressure and perfusion signals have been obtained and the relationship between intracuff pressure and cuff-trachea contact pressure analysed. It is shown that intracuff pressure may not accurately represent true contact pressure due to variations in the shape of the trachea, with the FBG contact pressure being larger than intracuff pressure, except when the sensor is positioned towards the more elastic posterior of the trachea. Furthermore, this paper has shown that PPG signals can reliably be obtained at the trachea and in the future could be used with cuff contact pressure to assess tracheal ischemia, which can result in injury. The measurements also have the potential to be able to identify when a good seal has been obtained and therefore could have a role in effective ventilation and reducing VAP caused by microaspirations. Cuff contact pressure has also been shown to vary during ventilation cycles, which could provide clinically useful information, such as the response the lungs and the trachea have to the ventilator pressure. The ability of iTraXS to monitor core vital signs has also been observed, with the possibility for the implementation into broader healthcare. The relatively simple addition of further optical sensors into iTraXS, would allow the measurement of other physiological parameters such as blood oxygenation, capillary refill time and core temperature.

## Data Availability

Data underlying the results presented in this paper are not publicly available at this time but may be obtained from the authors upon reasonable request.
